# Differential Degradation and Detoxification of an Aromatic Pollutant by Two Different Peroxidases

**DOI:** 10.3390/biom7010031

**Published:** 2017-03-18

**Authors:** Aysha Hamad Alneyadi, Iltaf Shah, Synan F. AbuQamar, Syed Salman Ashraf

**Affiliations:** 1 Department of Biology, United Arab Emirates University, P.O. BOX 15551, Al-Ain, UAE; 200907889@uaeu.ac.ae (A.H.A.); sabuqamar@uaeu.ac.ae (S.F.A.); 2 Department of Chemistry, United Arab Emirates University, P.O. BOX 15551, Al-Ain, UAE; altafshah@uaeu.ac.ae

**Keywords:** organic pollutants, enzymatic remediation, Sulforhodamine B, soybean peroxidase, chloroperoxidase

## Abstract

Enzymatic degradation of organic pollutants is a new and promising remediation approach. Peroxidases are one of the most commonly used classes of enzymes to degrade organic pollutants. However, it is generally assumed that all peroxidases behave similarly and produce similar degradation products. In this study, we conducted detailed studies of the degradation of a model aromatic pollutant, Sulforhodamine B dye (SRB dye), using two peroxidases—soybean peroxidase (SBP) and chloroperoxidase (CPO). Our results show that these two related enzymes had different optimum conditions (pH, temperature, H_2_O_2_ concentration, etc.) for efficiently degrading SRB dye. High-performance liquid chromatography and liquid chromatography –mass spectrometry analyses confirmed that both SBP and CPO transformed the SRB dye into low molecular weight intermediates. While most of the intermediates produced by the two enzymes were the same, the CPO treatment produced at least one different intermediate. Furthermore, toxicological evaluation using lettuce (*Lactuca sativa*) seeds demonstrated that the SBP-based treatment was able to eliminate the phytotoxicity of SRB dye, but the CPO-based treatment did not. Our results show, for the first time, that while both of these related enzymes can be used to efficiently degrade organic pollutants, they have different optimum reaction conditions and may not be equally efficient in detoxification of organic pollutants.

## 1. Introduction

Organic compounds are a major class of pollutants that are frequently detected in different water bodies [1]. These compounds, including some that are potentially carcinogenic, are becoming a serious environmental issue and pose serious health risks to humans and other aquatic organisms [2,3,4]. Organic pollutants include pesticides, industrial waste, pharmaceuticals, and personal care products (PPCPs). Industrial organic waste, especially aromatic textile dyes, are considered one of the most abundant and hazardous pollutants. For example, some classes of dyes, such as azo dyes, are mutagenic and are linked to increasing incidences of cancer amongst textile workers [5,6]. In addition to their potentially carcinogenic nature, these dyes can reduce sunlight penetration and dissolved oxygen in water bodies.

**Figure 1:**
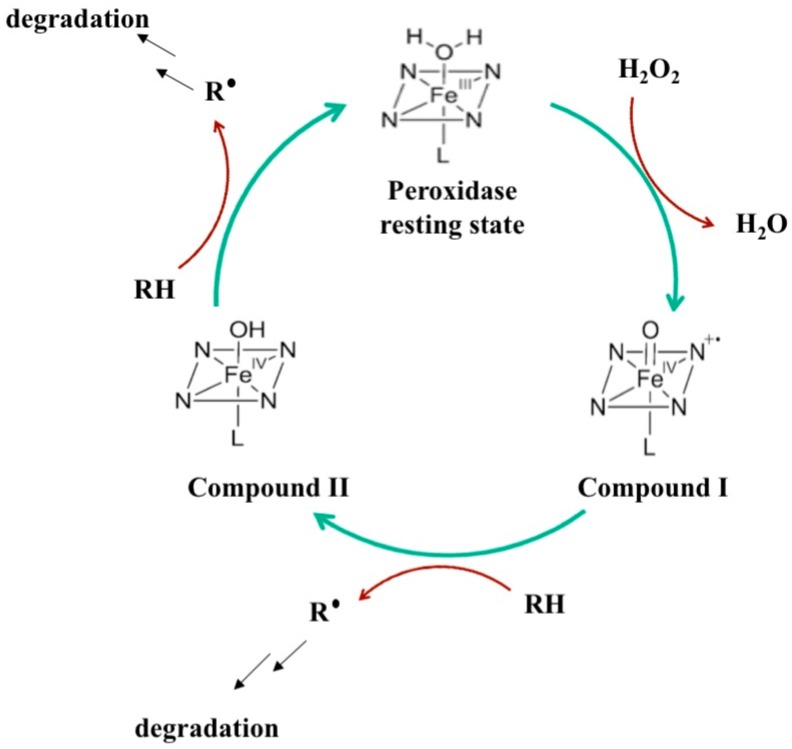
The catalytic cycle of heme-peroxidases. RH: organic pollutant; R^●^: radical form of the organic pollutant.

Chemical, physical, and biological approaches have all been used to efficiently degrade organic pollutants [7,8,9,10,11,12,13]. However, biological methods are increasingly attracting the attention of scientists as they are more environmentally benign than other strategies [14,15,16,17,18]. In biological methods, agents such as microorganisms, plants or enzymes from different origins are used to degrade different classes of organic pollutants [19,20,21,22]. Enzymatic treatments, particularly peroxidases, offer the added advantage of scalability, especially when coupled with recombinant DNA technologies. A review of the literature shows that diverse peroxidases, such as soybean (SBP), manganese, lignin, chloroperoxidase (CPO), and horseradish (HRP) peroxidases have all been extensively used to efficiently degrade various classes of organic pollutants [23,24,25,26,27,28]. All of these peroxidases are heme-containing enzymes and belong to the same superfamily of heme-dependent peroxidase (E.C. 1.11.1) and hence have a conserved catalytic cycle (Figure 1) that allow them to oxidize a wide range of substrates or pollutants. Briefly, the catalytic cycle starts with the resting form of the enzyme (with the heme iron in Fe(III)^+^ state) reacting with hydrogen peroxide to form a Fe(IV^+●^) cation radical form of the enzyme (called Compound **I**), which further reacts with its substrate (or pollutant, RH), to form a Fe(IV^+^) form called Compound **II** and a pollutant radical. Compound **II** further reacts with another molecule of the pollutant to return to the resting form of the enzyme and generate another pollutant radical. These pollutant radicals can further react with themselves or other species in the reaction mixture leading to further degradation.

The promiscuity of these peroxidases with regards to the diverse substrates they can act upon and the common catalytic cycle has led to the impression that all peroxidases are same/similar when it comes to the degradation of organic pollutants. Although plausible, this hypothesis has not been thoroughly tested. Despite the numerous reports of various peroxidases being used for the degradation of organic pollutants, only a few studies have been published that highlight differences between these enzymes in terms of their specificities and degradation mechanisms/pathways. For example, Li et al. reported that two closely related peroxidases, SBP and HRP, were both able to degrade triclosan [26]. However, in their study, SBP, but not HRP, was able to completely eliminate the toxicity of triclosan. We have also recently carried out a comparative study of the degradation of two different thiazole compounds using SBP and CPO enzymes. Our results showed that SBP could efficiently degrade Thioflavin T (ThT), while CPO only produced a chlorinated form of ThT without any actual degradation [27]. Additionally, SBP-degraded ThT was non-phytotoxic, whereas CPO-treated ThT was still toxic to *Lactuca sativa* seeds (data not shown). In the present study, we have extended our previous study [27] by using these two related and commonly used peroxidases (SBP and CPO) to examine in detail the degradation of another aromatic dye, Sulforhodamine B (SRB dye). Although not a typical organic pollutant, Sulforhodamine B is a carcinogenic dye and has been used by scientists in the field as a model aromatic pollutant to study non-enzymatic oxidative degradation as well as adsorption from simulated waste water [29,30,31,32]. In addition to studying enzymatic degradation of SRB dye, we tested the phytotoxicity of the intermediates generated during the remediation process by the two enzymes. Our results show that these two peroxidases have different optimum operating conditions with regards to SRB dye degradation and produce different degradation products with different toxicities.

## 2. Results and Discussion

### 2.1. The Requirement for Redox Mediator

**Figure 2:**
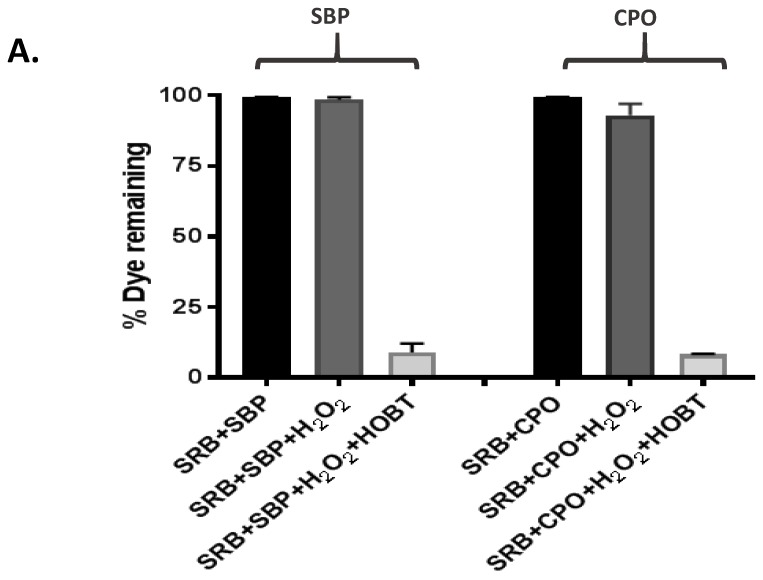

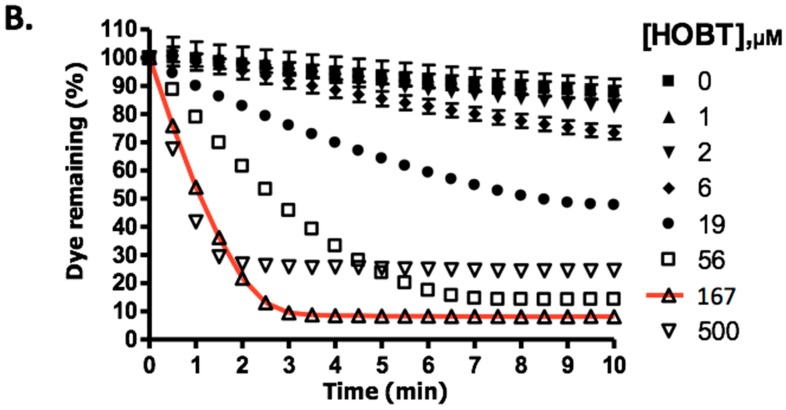
The effect of the redox mediator, 1-Hydroxybenzotriazole (HOBT) on Sulforhodamine B (SRB) dye degradation by Soybean peroxidase (SBP)and Chloroperoxidase (CPO). (**A**) SRB dye degradation by SBP in the absence and presence of 100 μM HOBT. [SBP] = 50 pM, [SRB dye] = 10 ppm, [H_2_O_2_] = 0.2 mM, pH = 4. (**B**) HOBT concentration optimization for CPO-based degradation for SRB dye. [CPO] = 440 pM, [SRB dye] = 6.25 ppm, [H_2_O_2_] = 0.2 mM, pH = 2.

It is well established that most peroxidases are able to oxidize a wide range of organic substrates in the presence of hydrogen peroxide. However, in some cases peroxidases also require a small, diffusible, and easily oxidized compound known as a “redox mediator” to facilitate the oxidation-reduction reaction [33,34]. Our initial degradation experiments with SRB dye showed neither SBP nor CPO were able to degrade the SRB dye at all in the presence of H_2_O_2_ alone (Figure 2A). However, when 1-hydroxybenzotriazole (HOBT), one of the most commonly used redox mediators, was added to the reaction mixture, rapid and efficient SRB dye degradation was observed (Figure 2A,B). This absolute requirement of HOBT was not observed in the SBP-degradation of many other dyes [27,35], but is consistent with peroxidase-mediated degradation of Crystal Ponceau 6R and ThT dyes [27,36]. Optimization of HOBT concentration for efficient SRB dye degradation showed that 50 μM HOBT was the optimum amount of redox mediator for SBP-based degradation, whereas the CPO-based reaction showed the best degradation of SRB dye at 167 μM HOBT (Figure 2B). Interestingly, higher concentrations of HOBT (beyond the optimum) caused a significant reduction in SRB dye degradation by both SBP and CPO. This was most likely because HOBT at higher concentrations may compete with SRB dye to bind to the active site of the enzymes, and hence lead to a slower degradation rate.

### 2.2. Effect of Reaction pH

**Figure 3:**
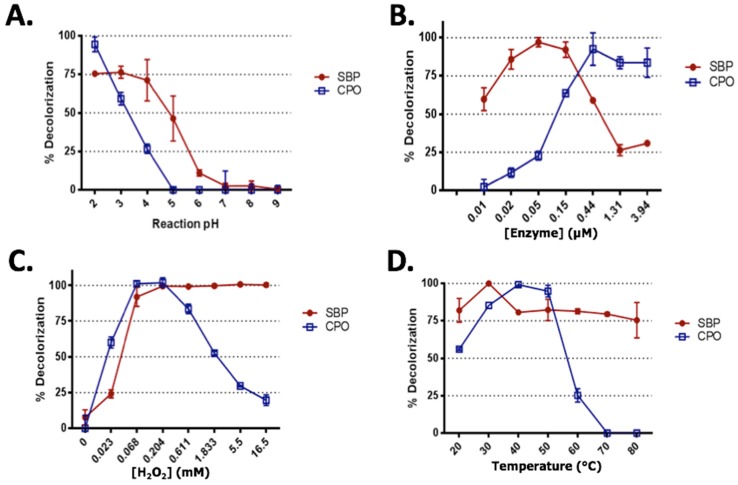
Optimization studies for SRB dye degradation by SBP and CPO. (**A**) pH profile ([SRB dye] = 15 ppm, [SBP/CPO] = 86 pM, [H_2_O_2_] = 0.2 mM, [HOBT] = 50 μM); (**B**) Enzyme concentration (([SRB dye] = 15 ppm, (SBP, pH) = 4, (CPO, pH) = 2 [H_2_O_2_] = 0.2 mM, [HOBT] = 50 μM); (**C**) H_2_O_2_ concentration (SBP: pH = 4, [SBP] = 50 pM, [SRB dye] = 10 ppm, [HOBT] = 50 μM; CPO: pH = 2, [CPO] = 440 pM, [SRB dye] = 6.25 ppm, [HOBT] = 167 μM); and (**D**) Effect of reaction temperatures of SRB dye degradation by SBP and CPO (SBP: pH = 4, [SBP] =17 pM, [SRB dye] = 10 ppm, [HOBT] = 16 μM, [H_2_O_2_] = 0.05 mM; CPO: pH = 2, [CPO] = 43 pM, [SRB dye] = 6.25 ppm, [H_2_O_2_] = 0.05 mM , [HOBT] = 82 μM).

Enzyme-based reactions are strongly influenced by the pH of the solution as changing the pH affects the ionization of the amino acid side chains in the active site of the enzyme, thus affecting the enzymatic activity. Changes in pH may also alter the ionization state of the substrates, which may also affect binding to the enzyme. Therefore, we examined the effect of pH on SBP- and CPO-mediated degradation of SRB dye, while keeping the other parameters constant. As expected, both enzymes were active under acidic conditions, and were totally inactive in the pH range 6–9. For SBP-based degradation of SRB dye, there were no significant differences in the decolorization percentages of SRB dye at pH values of 2, 3, or 4. However, SRB dye degradation by CPO was optimal at pH 2 and further increases in pH caused the degradation to decrease dramatically until it reached almost 25% at pH 4 (Figure 3A). These results are in agreement with previously published studies with SBP, where maximum degradation of Trypan Blue [35], Crystal Ponceau 6R [36], Amido Black [37], and Remazol Turquoise Blue G 133 [38] dyes were observed between pH 3–4, whereas CPO degradation of Alizarin Red and Crystal violet [39], Orange G and Sunset Yellow [40], and Thioflavin T [27], was maximum in the pH range between 2–3. Collectively, these results indicate that enzymatic remediation activity is significantly influenced by the pH of the reaction mixture, and SBP and CPO have different pH optima of pH 3–4 and pH 2–3, respectively.

### 2.3. Enzyme Concentrations

Enzyme concentration plays a significant role in enzymatic-based degradation. In addition, it is important to establish the minimum enzyme concentration that efficiently degrades the organic pollutant to ensure the enzymatic approach is economically viable. Our enzyme optimization experiments showed that SBP was more efficient at degrading SRB dye compared with CPO (Figure 3B). For example, at an enzyme concentration of 50 pM, SBP showed almost complete degradation of SRB dye; however, degradation was only 25% using the same concentration of CPO. As can be seen from Figure 3B, the optimum concentration of SBP was 50 pM, while the optimum CPO concentration was about ten times higher at 440 pM. It is worth noting that high concentrations of enzymes caused less efficient degradation, which might be due to the excess enzymes reacting with and consuming all of the hydrogen peroxide present in the reaction mixture, leading to a lower degradation of the substrates. The optimized enzyme concentrations in this study are slightly lower than those reported in other studies. For example, the optimum CPO concentration for the degradation of Alizarin Red, Crystal Violet, Orange G, and Sunset yellow has been reported as 150 nM, 30 nM, 250 nM, and 50 nM, respectively [39,40].

### 2.4. Hydrogen Peroxide Optimization

Hydrogen peroxide is essential for any peroxidase-based reaction since it acts as a co-substrate and generates the radical form of the enzyme “compound **I**”, which then reacts with organic substrate. However, excess H_2_O_2_ might lead to the formation of the Compound **III** form of the peroxidase (Fe-^3+^-OOH^●^), which may then lead to irreversible heme destruction and eventual loss of activity [41]. Conversely, low and sub-optimal concentrations of H_2_O_2_ might restrict the enzymatic activity of these enzymes and lead to poor degradation of organic pollutants. This was clearly observed in the case of CPO-mediated degradation of SRB dye. There was no observed SRB dye degradation in the absence of H_2_O_2_ and the degradation percentage increased with increasing H_2_O_2_ concentration until it reached maximum degradation at 0.2 mM H_2_O_2_. However, the CPO-mediated SRB dye degradation decreased dramatically with further increases in H_2_O_2_ concentration reaching only about 15% at 16.5 mM H_2_O_2_ (Figure 3C). A similar inhibitory effect of high H_2_O_2_ concentration on peroxidase activity has been reported previously [27,40]. Interestingly, SBP enzymes showed a different behavior, and appeared to be resistant to oxidative heme damage even at very high concentrations of H_2_O_2_. As can be seen from Figure 3C, there was no significant change in the degradation of the SRB dye by SBP at H_2_O_2_ concentrations ranging from 0.2 mM to 16.5 mM. These results, along with those previously reported, suggest that SBP is a remarkably stable enzyme, compared to CPO, as it can tolerate high concentrations of strong oxidants, such as H_2_O_2_ [35,36,42].

### 2.5. Effect of Temperature on SRB Dye Degradation

Temperature has a significant effect on enzyme-based reactions, such that increases in temperature initially enhance enzyme activity. However, after the reaction reaches the maximum activity, further increases in temperature begin to denature the enzyme, change the active site, impair substrate binding, and eventually reduce catalysis [43]. This was clearly observed in CPO-mediated degradation of SRB dye (Figure 3D), where the degradation increased from 55% to 90% with increasing temperature from 20 °C to 40 °C (Figure 3D). However, the degradation of SRB dye dramatically decreased to approximately 25% when the temperature increased to 60 °C. Interestingly, CPO did not show any degradation at temperatures beyond 60 °C, suggesting that CPO had completely denatured and become inactive. Surprisingly there were no significant differences in SBP-based degradation of the SRB dye when the temperature increased from 20 °C to 80 °C (Figure 3D).

These results suggest that SBP is a robust and a thermostable enzyme. This observation is consistent with previous studies, which showed that SBP can maintain its activity at temperatures up to 80 °C [44,45]. Thermal stability studies of the two peroxidases were also conducted by pre-heating the enzymes for 10 min at elevated temperatures (up to 80 °C) before assaying them for activity at room temperature (data not shown). These experiments showed very similar results and further confirmed that CPO and SBP have very different thermal stabilities. Collectively, optimization experiments revealed that SBP and CPO have different optimum conditions for degrading SRB dye, as summarized in Table 1.

### 2.6. LC–MS Analyses

**Figure 4:**
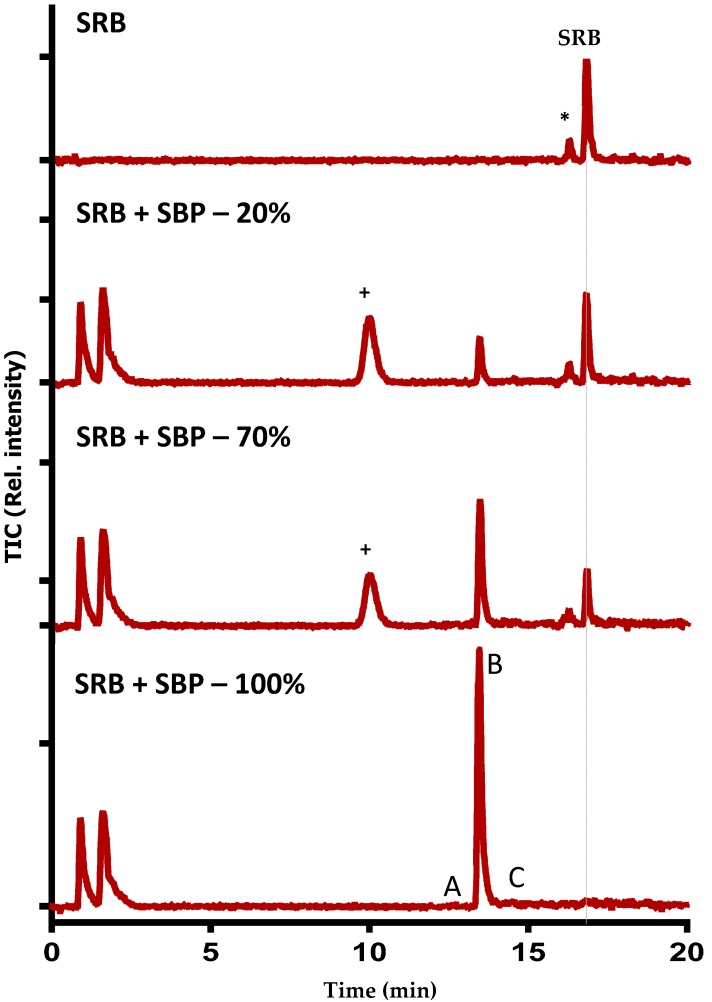
Liquid chromatography mass spectrometry (LC-MS) chromatogram of SRB dye degradation by SBP. “*” = De-ethylated form of SRB dye and “+” indicates the elution of HOBT. The conditions of 100% degradation are pH = 4, [SBP] = 50 pM, [SRB dye] = 10 ppm, [H_2_O_2_] = 0.2 mM, and [HOBT] = 50 μM. **A**–**C** are the compounds detected (Table 2). TIC: total ion chromatogram.

**Figure 5:**
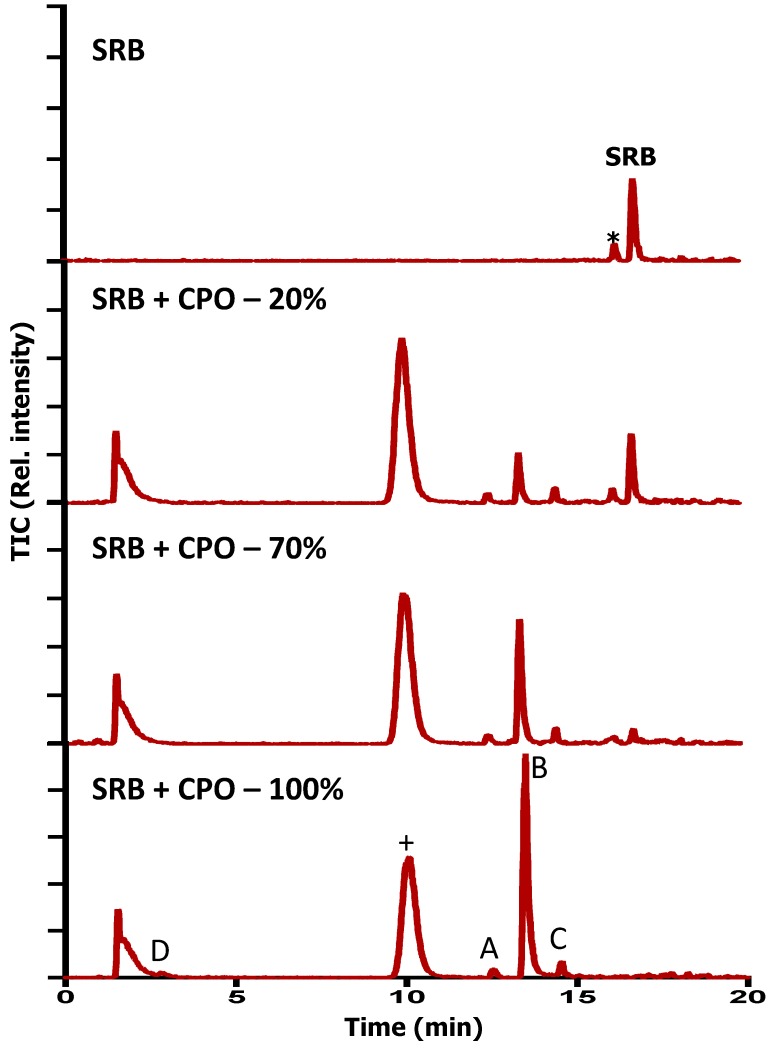
LC–MS chromatogram of SRB degradation by CPO. The conditions of 100% degradation are pH = 2, [CPO] = 440 pM, [SRB dye] = 6.25 ppm, [H_2_O_2_] = 0.2 mM, [HOBT] = 167 μM. **A**–**D** are the compounds detected (Table 2) and “+” indicates the elution of HOBT.

**Figure 6:**
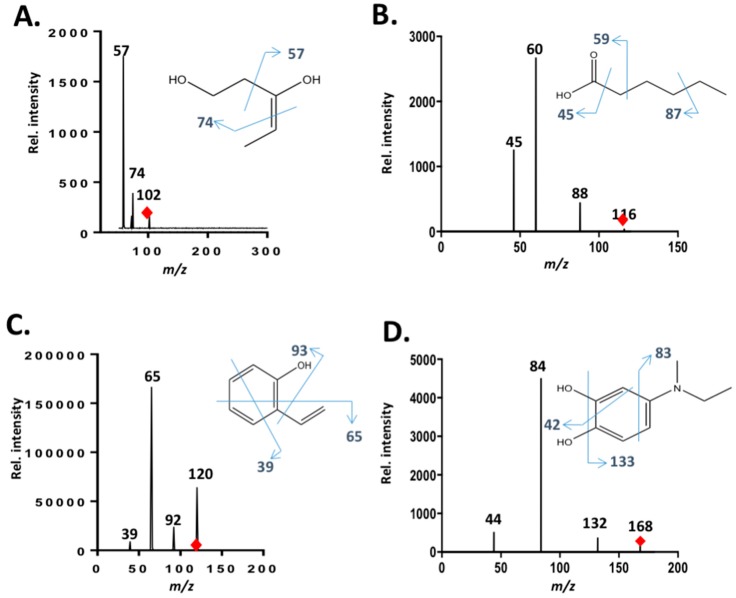
Tandem mass spectrometry fragmentation analyses of intermediates produced by after SRB dye degradation by SBP (Panels **A**, **B**, **C**) and CPO (Panel **D**). Panel (**A**) intermediate with *m*/*z* of 102; panel (**B**) intermediate with *m*/*z* of 116; panel (**C**) intermediate with *m*/*z* of 120 and panel (**D**) intermediate with *m*/*z* of 168.

To further confirm that dye decolorization was in fact due to the degradation of SRB dye, extensive liquid chromatography mass spectrometry analyses were conducted on pure and CPO- and SBP-degraded SRB dye samples. Figure 4 and Figure 5 show the total ion chromatograms of various SBP- and CPO-degraded SRB dye samples (pure dye, 20%, 70%, and 100% degradation). As can be seen from the chromatogram (Figure 4) of pure dye, there is a major peak at 559 *m*/*z*, which represents SRB dye. In addition, there is another peak at 531 *m*/*z*, which corresponds to the de-ethylated form of SRB dye (a minor contaminant). Addition of peroxidase, H_2_O_2_, and HOBT resulted in new peaks (intermediates) appearing until the SRB dye peak completely disappeared. Tandem mass spectrometry fragmentation experiments were conducted to further identify the structures of the intermediates produced after 100% degradation by either SBP or CPO (Figure 6). Interestingly, most of the intermediates detected (102 *m*/*z*, 116 *m*/*z*, and 120 *m*/*z*) were produced by both enzymes, however, their intensities (concentrations) in the SBP-based treatment were much lower compared to those in the CPO-based treatment, as shown in Table 2. It is quite likely that the differences observed in the intensities of the intermediates produced could just be due to the differences in the kinetics and reaction rates in the two different enzyme systems. However, CPO treatment of SRB dye produced at least one different intermediate (168 *m*/*z*) (Figure 6D).

**Figure 7:**
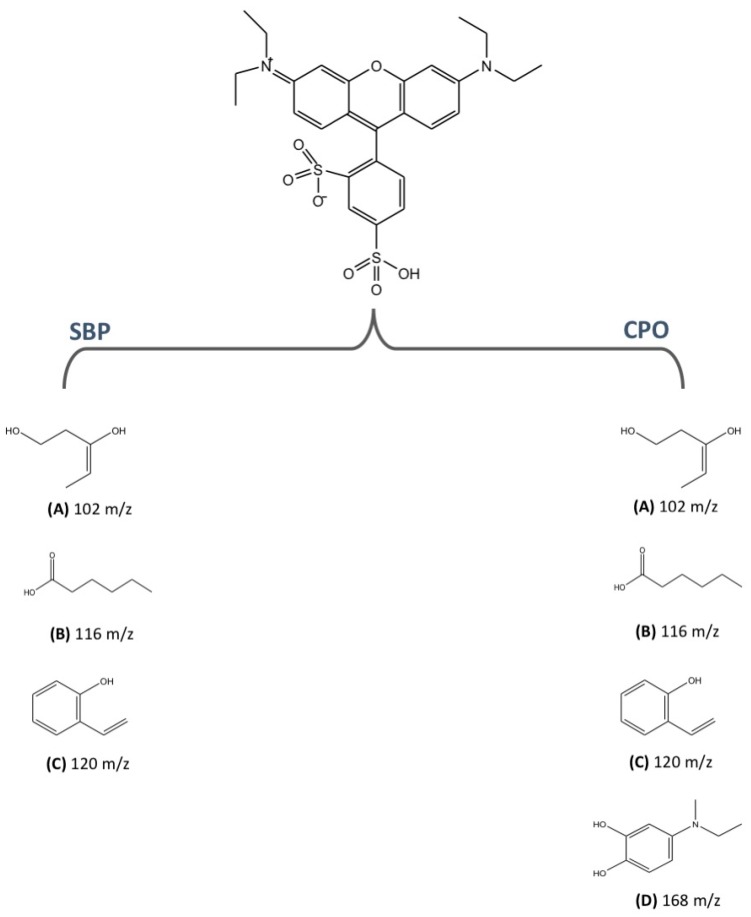
Proposed structures of some of the intermediates generated during SBP- and CPO-based degradation of SRB dye ((**A**–**C**) and (**A**–**D**), respectively).

Figure 7 summarizes the liquid chromatography tandem-mass spectrometry (LC-MS/MS) studies where the proposed structures of the intermediates generated during SRB dye degradation by SBP and CPO are shown. Our results concur with those of other studies of the photocatalytic degradation of Rhodamine B [46,47,48] and Triphenylmethane dyes, such as Crystal Violet [39], Malachite Green [49], and Basic Violet 3 [50], that report similar *N*-demethylation, desulfonation, and chromophoric ring openings during dye degradation.

### 2.7. Toxicity Tests

Most of the aromatic dyes (and other aromatic pollutants) can be toxic to various organisms [2,3,4], specifically to aquatic organisms and plants. As such, one of the aims of the enzymatic degradation process is a decrease or elimination of toxicity associated with the compounds being treated. This has been reported in several enzyme-based degradation studies, in which enzyme treatments significantly reduced the toxicity of the compounds [51,52]. To test this hypothesis in our system, phytotoxicity of untreated SRB dye and SRB dye that was enzymatically treated was studied using lettuce (*L. sativa*) seeds. Complete degradation of the treated SRB was confirmed by spectrophotometer and LC–MS analyses. Additionally, the other compounds (H_2_O_2_, HOBT and buffer) were tested alone and the result showed that they do not have a significant toxic effect.

**Figure 8:**
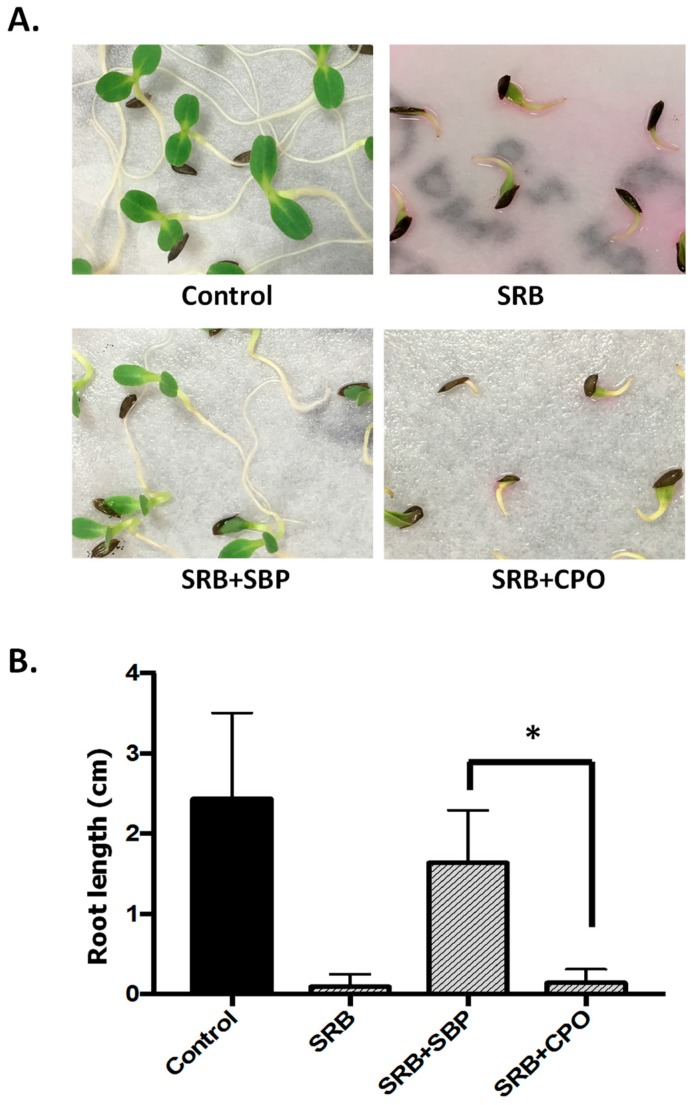
SRB dye toxicity on *Lactuca sativa* seed. (**A**) Representative sample of *L. stavia* seed treated by SRB dye (10 ppm), SRB dye decolorized by SBP (SRB + SBP) and SRB dye treated by CPO (SRB + CPO); (**B**) Root length (cm) after treating the seeds with SRB dye samples. Statistical analysis was performed using unpaired *t*-test (*n* = 80). The asterisk (*) shows a significant difference (*p* < 0.05).

Our data showed that SRB dye was toxic to the plant and significantly affected its growth and development. Surprisingly, there were no significant toxic effects of SRB dye on the actual germination of the seeds (Figure 8). Figure 8B also shows that after the SBP/H_2_O_2_ treatment, the toxicity of SRB dye was reduced. Surprisingly, treatment of SRB dye with CPO/H_2_O_2_ failed to eliminate the toxicity of the dye. Statistical analyses showed that there was no significant difference in the root length of seeds treated with SBP-degraded SRB dye (1.64 ± 0.66 cm) compared with the control (2.36 ± 1.12 cm), suggesting almost complete elimination of SRB dye toxicity to *L. sativa* seeds. Conversely, the mean root length of the seeds treated with CPO-degraded SRB dye was only 0.14 ± 0.17 cm compared to those of seeds treated with SBP-degraded SRB dye (Figure 8B). This difference between SBP- and CPO-treated SRB dye shows the importance of phytotoxicity analyses of remediated wastewater samples. One possible reason for the difference could be due to the way the two enzymes degraded SRB dye. Figure 7 and Table 2 show that, although most of the intermediates produced by both enzymes were the same (compounds **A**, **B**, and **C**), the concentrations of intermediates A and B after CPO-based degradation were significantly higher than those produced in the SBP-based treatment. This difference might explain the toxicity of the CPO-treated samples. Additionally, the 168 *m*/*z* intermediate (compound **D**) was only observed during the CPO treatment, so it is possible that the toxicity of the CPO-treated SRB dye could also be partly due to this compound. Interestingly, other researchers have also reported that enzymatic degradation of dyes could result in increased toxicity in some cases [53,54]. These results highlight the dramatic differences between SBP and CPO enzymes and also stress the importance of toxicological evaluation of peroxidase-degraded enzymatic pollutants.

### 2.8. The Effect of Halides on Enzymatic Degradation

**Figure 9:**
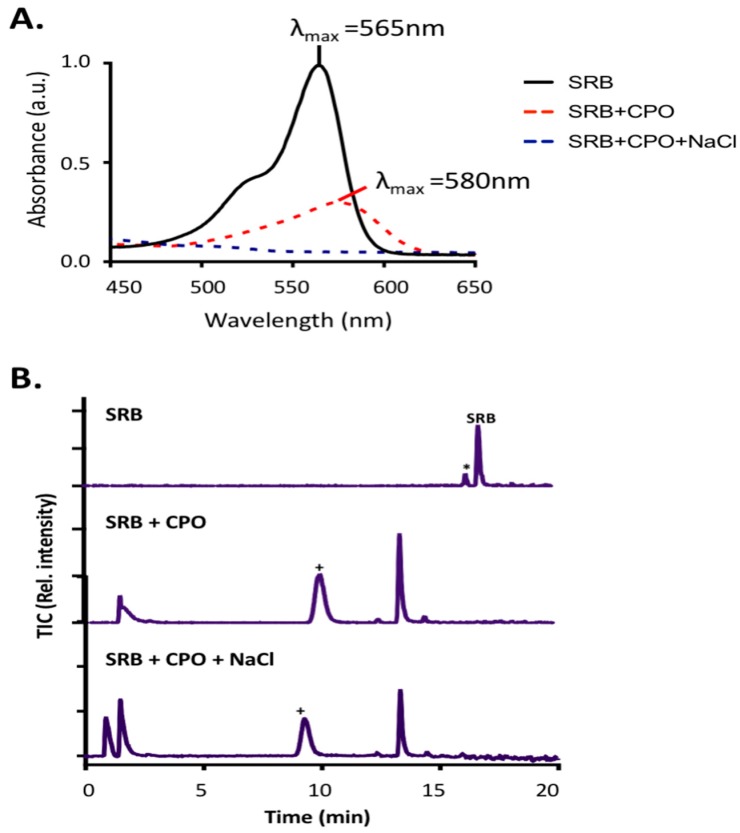
The effect of halide on CPO-based degradation of SRB dye. (**A**) UV-Vis chromatograms and (**B**) Total ion chromatograms of SRB dye degradation by CPO in the absence and presence of NaCl.

**Figure 10:**
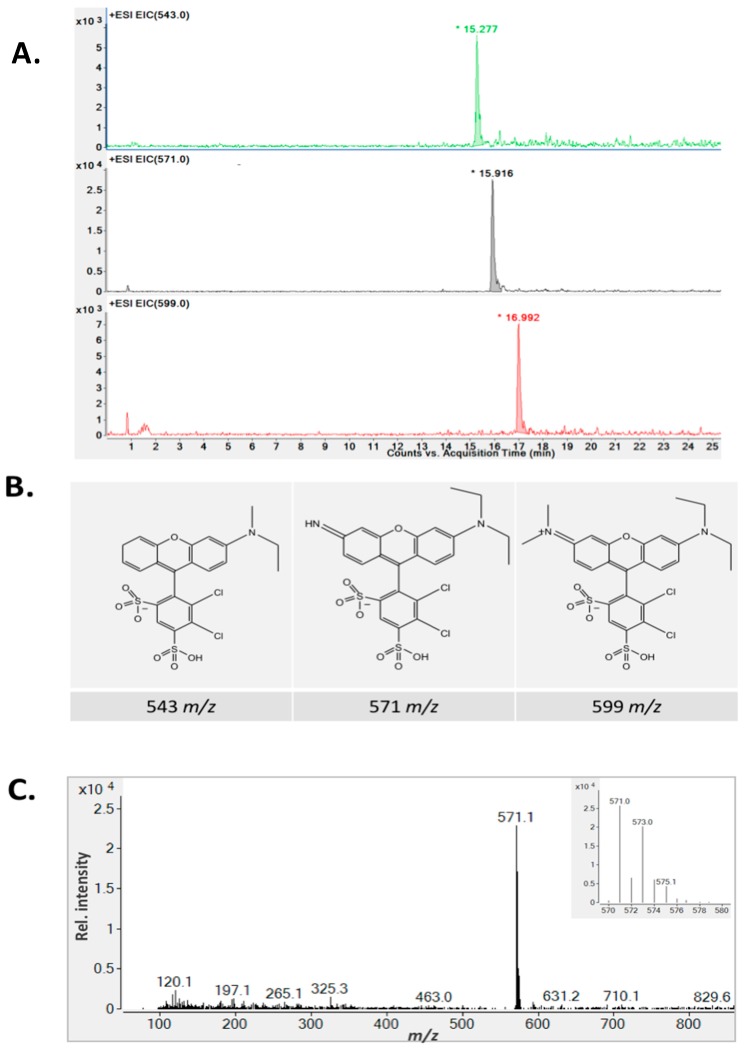
LC–MS analyses of chlorinated intermediates. (**A**) Extracted ion chromatogram of chlorinated intermediates produced by CPO in the presence of NaCl (33 mM); (**B**) The proposed structure of the chlorinated intermediates; (**C**) MS spectra at 15.87 min showing the 571 *m*/*z* intermediates. The insert shows the chlorine signature pattern for dichlorination.

Some peroxidases, especially CPO, can halogenate aromatic compounds in the presence of halides [55]. Therefore, we also investigated the effect of chloride ions in SBP- and CPO-mediated degradation of SRB dye. As shown in Figure 9, the presence of chloride ions did not have any significant effect on SBP-mediated degradation of SRB dye. However, the addition of chloride ions to the CPO reaction mixture resulted in a dramatic shift in the λ_max_ of SRB dye from 565 nm to 600 nm, indicating possible formation of (a) new compound(s) (Figure 9). Further investigation using LC–MS showed that the CPO-based treatment in the presence and absence of chloride ions resulted in some of the same intermediates being produced. However, some new chlorinated intermediates were detected in the samples that included chloride ions in the reaction mixture (Figure 10A). The proposed structures of the chlorinated intermediates (based on LC–MS/MS analyses) are shown in Figure 10B. Additionally, the presence of dichlorinated intermediates was further confirmed by the characteristic chlorine isotope signatures ^35^Cl and ^37^Cl. For example, the 571 *m*/*z* intermediate showed several lines, which could be interpreted as a combination of chlorine isotopes representing M+, M+2, and M+4, as illustrated in Figure 10B. Chlorination of organic compounds by CPO has also recently been reported in CPO-mediated treatment of ThT, in which addition of Cl to the original compound made it colorless, but did not result in any actual degradation of the compound [27]. However, this chlorination property of CPO is not extensively reported and, to our knowledge, chlorination of SRB dye (and ThT in a previous study [27]) are the first reports of dyes being halogenated by CPO.

## 3. Materials and Methods

### 3.1. Enzymes and Chemicals

Sulforhodamine B at 97% purity (molecular formula = C_27_H_30_N_2_O_7_S_2_, formula weight = 558.6 g·mol^−1^) was purchased from AnaSpec (Fremont, CA, USA). SBP and CPO were purchased from Bio-Research Products (North Liberty, IA, USA). The specific activities of the enzymes were as follows: SBP was 2700 IU/mg (1 mg/mL), 26 μM, while CPO activity was 1296 IU/mg (17 mg/mL), 405 μM. The enzymes were diluted in water to the appropriate concentrations as needed. Hydrogen peroxide (30% *w*/*v*) was purchased from Sigma-Aldrich (St. Louis, MO, USA). Universal buffers were prepared using 0.1 M citric acid and 0.2 M K_2_HPO_4_. LC–MS grade acetonitrile, methanol, and formic acid were obtained from Sigma-Aldrich Chemical Co.

### 3.2. Dye Degradation and Optimization Studies

The dye degradation assays were carried out in 3 mL total volume by incubating the specific concentration of the dye with either SBP or CPO in the appropriate buffer (pH 4 for SBP and pH 2 for CPO) and different concentrations of hydrogen peroxide and monitored using a 4 mL quartz cuvette in a Spectrophotometer Carry 60 (Agilent Technologies, ‎Santa Clara, CA, USA) as previously described [35,36]. When needed, the redox mediator, HOBT, was added in the reaction mixture to help with the dye degradation assay. The exact experimental conditions and reagent concentrations are also included in all the figure legends. Different experiments were conducted to optimize the reaction conditions of HOBT concentration, buffer pH, enzymes concentrations, H_2_O_2_ concentration, and reaction temperature using one-factor-at-a-time methodology. That is, the effect of each factor was investigated by changing one factor while keeping the other parameters constant. All the optimization experiments (except for temperature studies) were conducted using a 96-well plate in a BioTek Epoch plate reader (BioTek, Winooski, VT, USA) and monitoring the λ_max_ of SRB dye (λ_max_ = 565) for 10 min. The effect of temperature was studied by incubating the enzyme reaction mixture (enzyme, H_2_O_2_, dye, buffer, HOBT) at the specific temperature for 10 min, after which the spectra were recorded. In all experiments, % decolorization was calculated as follows: $\%\;\mathrm{decolorization}=\;\frac{A0-\mathrm{At}}{A0}\;\times \;100,$ where A_0_ refers to the initial absorbance of dye solutions and A_t_ refers to the absorbance of the solutions at any given time. Each optimization experiment was replicated at least 3 times under identical conditions and the results are reported as mean ± SD (Standard Deviation).

### 3.3. HPLC and LC–MS Analyses

The biodegradation and intermediates produced after the decolorization of SRB dye by SBP and CPO were analyzed using LC–MS. All SRB dye samples were filtered using a 0.45 μm syringe filter prior to analysis. The LC–MS was fitted with a ZORBAX Extend-C18 column (3.5 μm particle size, 4.6 mm × 250 mm length, Agilent Technologies) maintained at 35 °C, coupled to a tunable Ultraviolet–visible (UV-Vis) detector (Agilent Technologies) and 6420 Triple Quadrupole LC–MS System detector (Agilent Technologies). The mobile phases were: A = water with 0.1% formic acid and B = 100% acetonitrile. The gradient was 0–5 min: 0% B, 5–20 min: 0%–100% B, 20–25 min: 100% B, and 25–30 min: 0% B at a 0.2 mL/min flow rate. The electrospray ionization source used in the LC–MS system was in positive polarity mode. Other LC–MS operating conditions were as follows: capillary voltage was set at 4 kV; the nebulizer pressure was 45 psi; drying gas flow was 11 L/min and drying temperature was 325 °C. The mass range monitored was 100–1000 Da. Tandem MS experiments were conducted using the product ion mode wherein nitrogen gas was used as a collision gas with different collision energies.

### 3.4. Phytotoxicity Assay

The toxicity of SRB dye before and after complete enzymatic degradation was measured using the phytotoxicity effect of the SRB dye samples on the germination of lettuce (*L. sativa*) seeds, as previously described [56] with slight modifications. Briefly, Whatman filter paper (No. 3, sterilized) *L. sativa* was placed on a Petri dish and saturated with 4 mL of the sample, avoiding bubble formation. Twenty seeds were placed on the filter paper with sufficient space to allow germination. Petri dishes were incubated for 5 days in a humidified chamber at 22 ± 2 °C to avoid moisture loss. Distilled water was used as a negative control and each sample was tested in quadruplicate. The effect of untreated and treated dye (completely degraded SRB dye) samples on germination was calculated according to the following equation:

$$\%\;\mathrm{germination}\;=\;\frac{\mathrm{number}\;\mathrm{of}\;\mathrm{germinated}\;\mathrm{seeds}}{\mathrm{total}\;\mathrm{number}\;\mathrm{of}\;\mathrm{seeds}}\;\times \;100$$

Complete degradation of the peroxidase treated SRB dye was confirmed by spectrophotometric as well as LC–MS analyses. Additionally, appropriate controls were also included, where all the reagents (HOBT, H_2_O_2_, etc.) were tested alone and no significant phytotoxicity was observed. Moreover, the effect of the original dye and the dye samples degraded by either SBP or CPO was also examined by measuring the length of the root of the germinated seeds and the percentage of root inhibition was calculated as below:

$$\%\;\mathrm{root}\;\mathrm{inhibition}=\;\frac{\mathrm{mean}\;\mathrm{of}\;\mathrm{control}\;\mathrm{root}\;\mathrm{growth}\;-\;\mathrm{mean}\;\mathrm{of}\;\mathrm{sample}\;\mathrm{root}\;\mathrm{growth}}{\mathrm{mean}\;\mathrm{of}\;\mathrm{control}\;\mathrm{root}\;\mathrm{growth}}\times \;100$$

Statistical analyses were conducted for each group of treated seeds (*n* = 80). The data were analyzed via unpaired *t*-tests. Data are reported as group mean ± SD and significance for all statistical comparisons was set at *p* < 0.05.

## 4. Conclusions

In summary, the data show that peroxidases are a versatile group of enzymes that have potential for organic pollutant degradation and wastewater remediation. However, these peroxidase-based treatments can be affected by various factors, such as the type of peroxidase, pH, temperature, dye structure, and the presence of redox mediators. All these factors must be optimized for efficient degradation. Interestingly, we also showed that related peroxidases (from same superfamily), such as SBP and CPO, behaved differently when degrading SRB dye, with different pH optima, thermal stabilities, and H_2_O_2_ tolerances. Furthermore, LC–MS studies showed that the treatment of SRB dye with these two enzymes generated some different products. While SBP-mediated degradation of SRB dye completely eliminated the dye toxicity, the CPO treatment did not. This surprising result highlights the importance of detailed toxicological evaluations of the degraded organic pollutant. In conclusion, our results further emphasize that commonly used peroxidases may not all be “equal” when used for remediation of organic pollutants and have specific optimal conditions and may use different mechanisms and pathways for the degradation reaction.

## Figures and Tables

**Table 1:** Optimization experiments of SRB dye degradation by SPB and CPO.

Optimum Conditions	SBP-Based Degradation	CPO-Based Degradation
[HOBT] (μM)	50	167
pH	4	2
[Enzyme] (pM)	50	440
[Dye] (ppm)	10	6.25
[H_2_O_2_] (mM)	0.2	0.2
Operational temperature range (°C)	20–80	20–50

**Table 2:** Intensity of intermediates produced during the degradation of SRB dye by SBP and CPO.

Compound ID	*m*/*z*	RT (min)	SRB DYE + SBP		SRB DYE + CPO	
				Intensity		Intensity
A	102	12.56	+	0.5 × 10^4^	+	4.0 × 10^4^
B	116	14.56	+	1.0 × 10^4^	+	6.0 × 10^4^
C	120	13.46	+	7.5 × 10^4^	+	9.0 × 10^4^
D	168	2.84	−	−	+	1.4 × 10^4^

RT: retention time; +: compound detected; -: compound not detected.
